# 3D MRI flow analysis in an in-vitro system modelling continuous left ventricular support: effect of cannula position in the thoracic aorta

**DOI:** 10.1186/1532-429X-14-S1-W29

**Published:** 2012-02-01

**Authors:** Christoph Benk, Ramona Lorenz, Friedhelm Beyersdorf, Rolf Klemm, Maximilian Russe, Philipp Blanke, Jan G Korvink, Michael Markl

**Affiliations:** 1 Department of Cardiovascular Surgery, University Hospital Freiburg, Freiburg, Germany; 2 Department of Radiology, Medical Physics, University Hospital Freiburg, Freiburg, Germany; 3 Department of Radiology, University Hospital Freiburg, Freiburg, Germany; 4 IMTEK - Institute of Microsystem Technology, Laboratory for Simulation, University Freiburg, Freiburg, Germany; 5 Department of Radiology and Biomedical Engineering, Northwestern University, Chicago, Chicago, IL, USA; 6 FRIAS, Freiburg Institute of Advanced Studies, Freiburg, Germany

## Background

Continuous blood flow from left ventricular assist devices (LVAD) has become an important treatment option for heart failure patients. However, recent reports indicate that the complex geometric alterations of the vascular system associated with LVAD implantation as well as the introduction of constant flow into the aorta may alter blood flow patterns. These can potentially affect perfusion patterns to the supra-aortic vessels or cause structural changes to the aortic root leading to regurgitation and valve dysfunction. The purpose of this study was to evaluate the feasibility of flow-sensitive 4D MRI for the investigation of such blood flow alterations in an in-vitro model simulating 3D blood in a physiological model of the left ventricle, aortic valve and aorta.

## Methods

In a MRI compatible mock loop circuit consisting of a silicon model of an aorta connected to a MEDOS ventricle to simulate the pulsatile flow of the native heart, three different outflow cannula positions (B 45° asc. aorta, C 90° asc. aorta and D desc. aorta) and a control model A (without LVAD) were tested. Time-resolved 3D phase contrast MRI with 3D velocity encoding (flow-sensitive 4D MRI, spatial resolution ~2.0-2.5mm, temporal resolution = 42.4ms) was applied to assess 3D aortic hemodynamics in all three models for a fully ejecting VAD and a reduced cardiac output (CO). 3D blood flow visualization using time resolved pathlines (EnSight, CEI, USA) was employed; blood flow velocities and flow volume were quantified in manually positioned 2D analysis planes using home built software (Matlab, USA).

## Results

**Figure 1:**
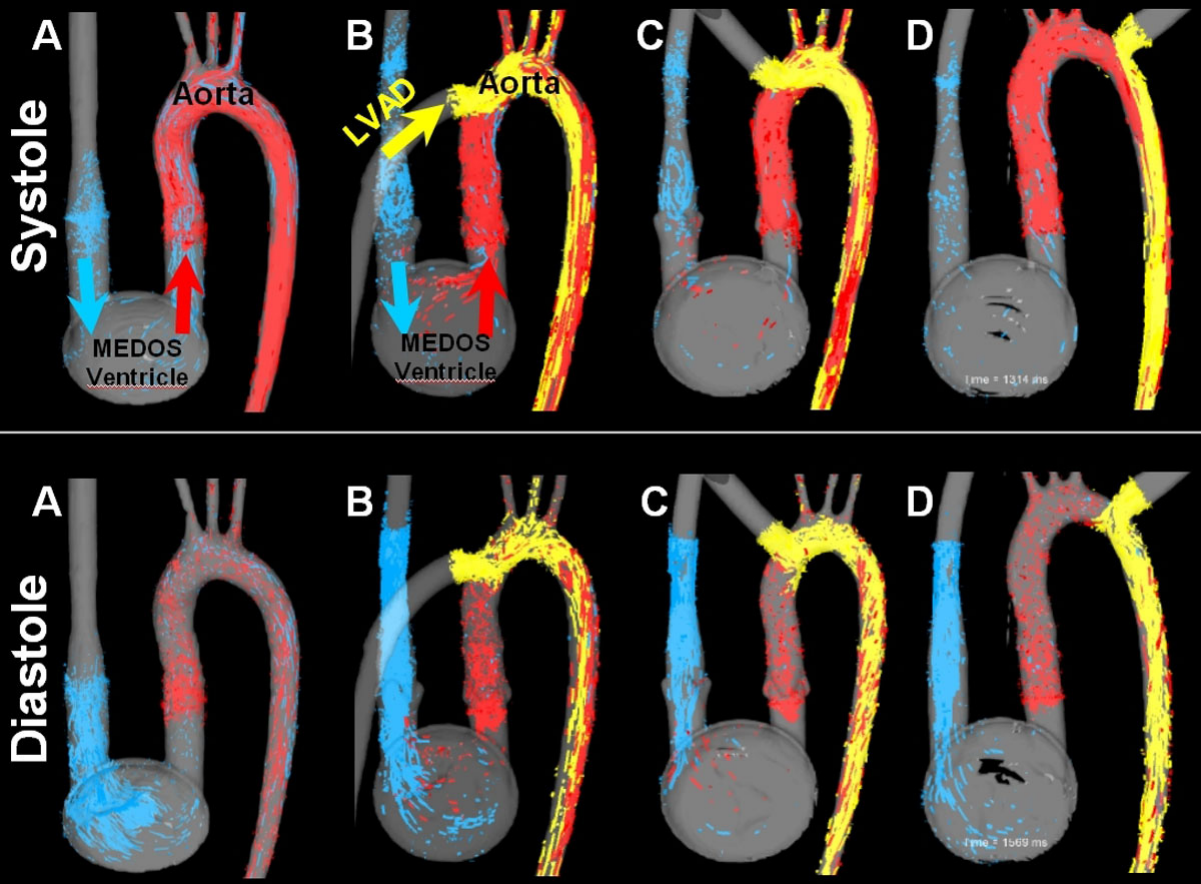
3D flow visualization in the four different models (A - D) during systole and diastole. Note the different systolic and diastolic filling patterns of the supra-aortic vessels for the different connections and maintained diastolic forward flow during LVAD support (B-D) compared to the aorta (A)

**Figure 2:**
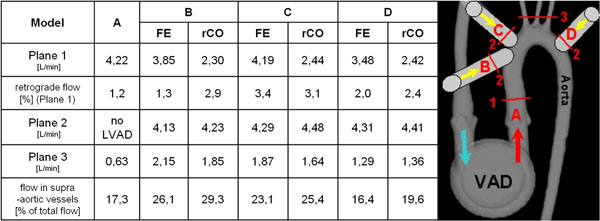
Blood flow velocities in analysis planes in all models under full ejecting (FE) heart and with reduced CO.

Blood flow patterns in the aorta illustrated the changes in hemodynamic due to LVAD inflow at different positions (figure 1). Compared to A, cannula position at the asc. aorta (B,C) led to increase in supra-aortic flow whereas D showed decreased supra-aortic flow. Interestingly, the presence and amount of retrograde flow into the asc. aorta and through the valve did not change (figure 2). Reduced CO did not lead to an increase in retrograde flow indicating that even limited cardiac function is sufficient to maintain forward flow and protect the aortic valve.

## Conclusions

Time-resolved 3D phase contrast MRI is feasible to model blood flow alterations in the aorta caused by the LVAD. Our preliminary results indicate that the cannula position may influence perfusion of the supra-aortic but has little effect on retrograde flow in the ascending aorta. Future experiments will focus on a more detailed evaluation of the effect on CO on to provide a better understanding of the impact of LAVD support on valve function.

